# Increased Level of α2,6-Sialylated Glycans on HaCaT Cells Induced by Titanium Dioxide Nanoparticles under UV Radiation

**DOI:** 10.3390/nano8040253

**Published:** 2018-04-19

**Authors:** Yuanyuan Ren, Xin Liu, Runqing Geng, Qunwei Lu, Rong Rao, Xi Tan, Xiangliang Yang, Wei Liu

**Affiliations:** 1 College of Life Science and Technology, Huazhong University of Science and Technology, Wuhan 430074, China; renyuanyuan@mail.hust.edu.cn (Y.R.); raorong_2004@126.com (R.R.); xtan@hust.edu.cn (X.T.); yangxl@hust.edu.cn (X.Y.); 2 Britton Chance Center for Biomedical Photonics at Wuhan National Laboratory for Optoelectronics, Hubei Bioinformatics & Molecular Imaging Key Laboratory, Systems Biology Theme, Department of Biomedical Engineering, College of Life Science and Technology, Huazhong University of Science and Technology, Wuhan 430074, China; xliu@mail.hust.edu.cn; 3 Laboratory of Molecular Biophysics of Ministry of Education, College of Life Science and Technology, Center for Human Genome Research, Huazhong University of Science and Technology, Wuhan 430074, China; runqinggeng@hust.edu.cn; 4 National Engineering Research Center for Nanomedicine, Huazhong University of Science and Technology, Wuhan 430074, China

**Keywords:** TiO_2_ nanoparticles, phototoxicity, sialic acids, reactive oxygen species, vitamin C

## Abstract

As one of the most widely used nanomaterials, the safety of nano-TiO_2_ for human beings has raised concern in recent years. Sialylation is an important glycosylation modification that plays a critical role in signal transduction, apoptosis, and tumor metastasis. The aim of this work was to investigate the cytotoxicity and phototoxicity of nano-TiO_2_ with different crystalline phases for human skin keratinocytes (HaCaT cells) under ultraviolet (UV) irradiation and detect sialic acid alterations. The results showed that the mixture of crystalline P25 had the highest cytotoxicity and phototoxicity, followed by pure anatase A25, whereas pure rutile R25 had the lowest cytotoxicity and phototoxicity. A25 and R25 had no effects on the expression of sialic acids on HaCaT cells. However, HaCaT cells treated with P25 and UV showed an increased level of alterations in α2,6-linked sialic acids, which was related to the level of reactive oxygen species (ROS) generated by nano-TiO_2_ and UV. The abundance of α2,6-linked sialic acids increased as ROS production increased, and vice versa. Antioxidant vitamin C (VC) reversed the abnormal expression of α2,6-linked sialic acids caused by nano-TiO_2_ and protected cells by eliminating ROS. These findings indicate that nano-TiO_2_ can alter the sialylation status of HaCaT cells under UV irradiation in a process mediated by ROS.

## 1. Introduction

Nanoparticles and nanotechnology are being developed rapidly and are increasingly encountered during the course of daily life. Due to the unique properties of titanium dioxide nanoparticles (nano-TiO_2_), including absorbing and scattering ultraviolet (UV) and photocatalysis, these nanoparticles have a variety of uses in many fields, such as additives in the cosmetics industry and whiteness enhancers in the paper industry [1,2]. However, nano-TiO_2_ have a high surface-to-volume ratio because of their nanoscale size, which may result in high surface energy and biological reactivity [3]. Studies of the potential health risk of nano-TiO_2_ for humans have revealed that the toxicity of nano-TiO_2_ is dependent on the size, shape, and crystalline phase of the particles, as well as their distribution in the body [4,5,6].

Due to the use of nano-TiO_2_ in paints, wastewater treatment, food products, and cosmetics, nano-TiO_2_ may come into contact with human skin and mucous membranes and thus enter the human body [7]. However, under UV irradiation, electrons in the nano-TiO_2_ valence band absorb photon energy and jump to the conduction band, leaving valence band holes that extract electrons from water or hydroxyl ions and generate reactive oxygen species (ROS), which are cytotoxic and genotoxic [8,9]. The phototoxicity and degree of damage associated with nano-TiO_2_ are dependent on the crystalline phase, size and concentration of the nanoparticles [4,10]. Nano-TiO_2_ can inhibit the growth of HaCaT cells via ROS generation and decrease the activity levels of enzymes, including superoxide dismutase (SOD), catalase (CAT), and glutathione peroxidase (GPx), under UV light [11]. ROS damage cells, tissues, and organisms by causing lipid peroxidation, altering the abundance of proteins, producing DNA mutations, and triggering apoptosis [12,13,14,15].

As previously reported, extensive studies have focused on how nano-TiO_2_ and UV disrupt and interfere with the processes of mRNA transcription and protein translation, but few studies have examined the effects of nano-TiO_2_ on post-translational modifications of proteins under UV light [16,17]. Glycosylation is an important post-translational modification. Advanced glycation end-products exist in the extracellular matrix of the dermis and the cell surface, where they influence healing of the skin, aging, elasticity and several skin disorders [18]. Keratinocytes, the main epidermal cells in the skin, were used in this study to investigate the effects of TiO_2_ nanoparticles on glycosylation.

The process of apoptosis is associated with alterations in glycans, including sialic acids, mannose, and fucose [19]. In cell lines of different histological origin (colon, breast, pancreas, and bladder cancer), as well as in normal human and mouse neutrophils, apoptosis is accompanied by the exposure of sugar chains recognized by the lectin from *Sambucus nigra* agglutinin (SNA), which is specific for siaα2,6Gal/GalNAc structures [20]. After induction of lymphocyte apoptosis, changes in 2,6-terminal sialic acids on the surface of apoptotic membrane blebs can either directly mediate cellular engulfment or enhance phagocytosis by cooperation with further engulfment signals [21]. Sialic acids are particularly abundant in mucins and at the epithelial cell surface, and they are usually the outermost monosaccharide of the glycocalyx.

Sialic acids are nine-carbon monosaccharides at the terminal position of oligosaccharides on the cell surface that are involved in cell metabolism [22], signal transduction [23,24], and tumor proliferation, invasion, and angiogenesis [25,26]. For example, influenza A viruses infect birds by recognizing α2,3-linked sialic acids on glycan chains, but they cannot recognize α2,6-linked sialic acids [27]. Nevertheless, the relationship between nano-TiO_2_ toxicity and alterations in sialylated glycans on the cell surface has not been studied.

In this study, the cytotoxicity and phototoxicity of different concentrations and crystalline phases of nano-TiO_2_ were investigated, and changes in sialylation on the surface of HaCaT cells caused by nano-TiO_2_ and UV were explored. Cells treated with UV and nano-TiO_2_ showed enhanced reactivity with *Sambucus nigra* agglutinin (SNA) and increased binding with α2,6-linked sialic acid. These changes were related to ROS generated by nano-TiO_2_ and UV, which led to changes in sialic acids. These findings suggest that sialic acid expression plays an important role in the toxic effects of nano-TiO_2_ on the skin. The results of this analysis offer insight into the toxicity of nano-TiO_2_ to guide the development of safety guidelines and future research into nanoparticle toxicity.

## 2. Materials and Methods

### 2.1. Materials

Nano-TiO_2_, an anatase-rutile mixture of nano-TiO_2_ P25 (Degussa Company, Essen, Germany), anatase nano-TiO_2_ (Sigma Company, St. Louis, MO, USA) and rutile nano-TiO_2_ (Macklin, Shanghai, China) were obtained from commercial sources. UV light was generated by an ultraviolet lamp (ZF-5, 365 nm, 8 W, 0.6 mW/cm^2^, Shanghai Huxi Instrument, Shanghai, China).

Fetal bovine serum (FBS), Dulbecco’s modified Eagle medium (DMEM), phosphate-buffered saline (PBS, pH 7.4), penicillin, streptomycin, and trypsin-EDTA were purchased from Gibco (Invitrogen, Carlsbad, CA, USA). 3-(4,5-Dimethylthiazol-2-yl)-2,5-diphenyltetrazolium bromide (MTT) and 2′,7′-dichlorofluorescin diacetate (DCFH-DA) were purchased from Sigma (St. Louis, MO, USA). *Sambucus nigra* lectin (SNA) labeled with fluoresceinIsothiocyanate (FITC) FITC and *Maackia amurensis* lectin I (MAL-I) were purchased from Vector labs (Burlingame, CA, USA).

Anhydrous dimethyl sulfoxide (DMSO) and vitamin C (VC) were purchased from Sigma Aldrich (St. Louis, MO, USA). ROSup was purchased from Beyotime (Shanghai, China).

### 2.2. Cell Culture

HaCaT cells, a spontaneously immortalized human keratinocyte cell line (ATCC), were cultured in 95% air and 5% CO_2_ at 37 °C in c supplemented with 10% FBS and 1% penicillin/streptomycin.

### 2.3. Preparation and Characterization of Nano-TiO_2_

The size and morphology of nano-TiO_2_ were visualized using transmission electron microscopy (TEM) (HT7700, Hitachi, Tokyo, Japan). The crystalline phase of nano-TiO_2_ P25 was detected by a Philips XPert PRO MPD X-ray diffractometer (Philips, Eindhoven, The Netherlands). Nano-TiO_2_ powder was sterilized by an autoclave, suspended in DMEM, and sonicated for 20 min in an ultrasonic bath. Before application to cells, the suspension was freshly prepared and immediately applied.

### 2.4. Cell Viability

Cells were seeded in 96-well-plates (Corning, Corning, NY, USA) at a density of 1 × 10^5^ cells per well and cultured for 24 h at 37 °C to reach 90% confluency. Cells were treated with 0, 10, 50, 100, 500, 1000 μg/mL of various crystalline phases of nano-TiO_2_ for 24, 48 and 72 h. After incubation, each well was washed with PBS three times and filled with DMEM containing 0.5 mg/mL MTT. The cells were incubated for 4 h at 37 °C, the supernatant was removed, DMSO was added to each well, and the cells were allowed to sit for 15 min. The plates were assessed at 492 nm using a microplate reader (Spectrafluor Plus, Tecan US, RTP, Durham, NC, USA).

### 2.5. Phototoxicity

Phototoxicity was assessed using MTT. Cells were seeded in 96-well plates and cultured for 24 h. The supernatant was replaced with different concentrations of nano-TiO_2_ P25 and cells were cultured for 21 h. The medium was replaced by PBS before UV radiation at 365 nm to avoid the effect of phenol red. After 1 h irradiation, fresh medium was added, and the cells were incubated for 2 h [7,28], after which cell viability was detected with the procedure described above.

### 2.6. Lectin Staining

HaCaT cells were cultivated in 6-well plates with sterilized coverslips to reach a density of 5 × 10^5^ cells per well. The cells were treated with 0 or 50 μg/mL nano-TiO_2_ P25 for 21 h, followed by UV irradiation for one hour. The cells were washed with cold PBS three times and fixed with 4% paraformaldehyde solution for 15 min. The fixed cells were incubated with 15 μg/mL FITC fluorescein-labeled lectins (SNA, MAL-I) for one hour in the dark at room temperature (RT) [29]. The cells were then observed under a laser confocal scanning microscope (Olympus, FV1000, Tokyo, Japan) to evaluate changes in sialylation. In order to compare sialic acid expression after various treatments, the fluorescence intensity of each pixel was quantified using NIH ImageJ software (National Institutes of Health, Bethesda, MD, USA).

### 2.7. Intracellular ROS Detection

DCFH-DA, a non-fluorescent, membrane-permeable compound, was used to detect intracellular ROS. After entering cells, DCFH-DA is rapidly de-acetylated to 2′,7′-dichlorodihydrofluorescein diacetate (DCFH), which can be converted by a broad range of ROS into 2,7-dichlorofluorescein (DCF), which is highly fluorescent and readily detected [30]. The fluorescence intensity was detected by flow cytometry and a fluorescence spectrophotometer at excitation and emission wavelengths of 485 and 530 nm, respectively. HaCaT cells were seeded in 12-well plates and cultured for 24 h. The cells were exposed to 0 or 50 μg/mL nano-TiO_2_ P25 for 3 h and washed twice with PBS, followed by exposure to pre-warmed PBS containing 20 μM DCFH-DA. The plates were treated with UV irradiation or left in the dark for one hour [28]. The cells were washed with cold PBS, trypsinized, centrifuged, and resuspended in 0.5 mL of cold PBS prior to measurement and flow cytometry analysis.

### 2.8. Statistical Analysis

All results are shown as the mean and standard deviation from at least three independent experiments. Statistical analyses were performed with Student’s two-tailed paired *t*-test. Values of *p* < 0.05 were considered statistically significant.

## 3. Results

### 3.1. Characterization of TiO_2_ Nanoparticles

**Figure 1:**
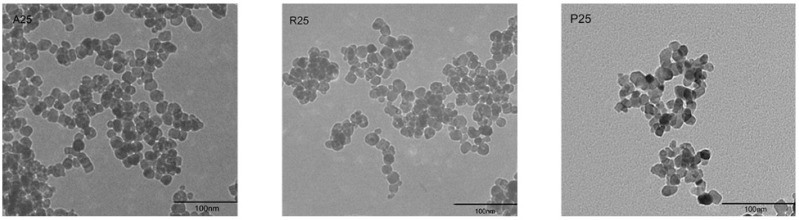
Morphological characterization of nano-TiO_2_ A25, R25, and P25 via TEM.

The morphology of nano-TiO_2_ was visualized under a transmission electron microscope (TEM) (Figure 1). The particle size was approximately 25 nm. Aggregation and large clusters were easily observed in all samples. The three tested crystalline phases of nano-TiO_2_ were A25, R25 and P25. X-ray diffraction (XRD) analysis showed that the crystalline composition of P25 was approximately 14% rutile and 86% anatase. R25 was pure rutile, whereas A25 was pure anatase (Table 1). The specific surface areas of the particles were similar among the nano-TiO_2_ samples.

### 3.2. Cytotoxicity and Phototoxicity of Nano-TiO_2_

**Figure 2:**
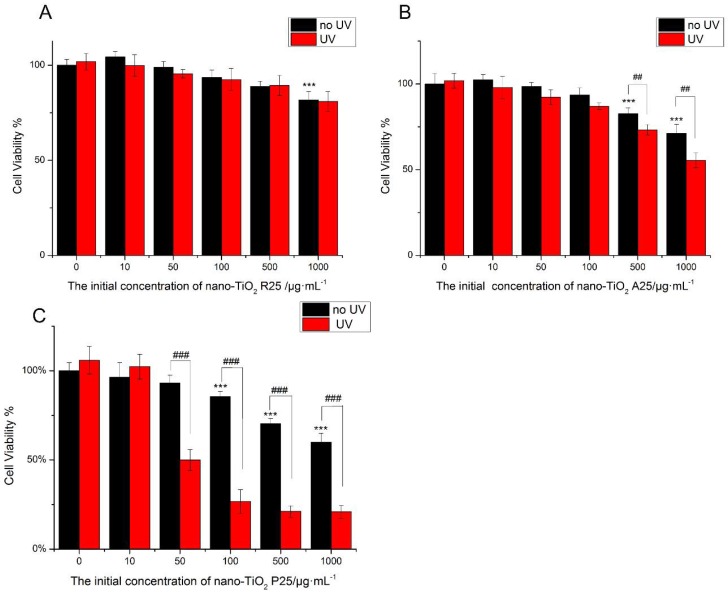
The effects of 24 h of exposure of human skin keratinocytes (HaCaT) cells to nano-TiO_2_ (**A**) R25, (**B**) A25, and (**C**) P25 with or without ultraviolet (UV) irradiation. Cell viability was quantified by 3-(4,5-Dimethylthiazol-2-yl)-2,5-diphenyltetrazolium bromide (MTT) assay. Significant differences are indicated where (*n* = 5) ± SEM, *** *p* < 0.001, ## *p* < 0.01, compared with the 0 μg/mL group. ### *p* < 0.001, compared with the same concentration of nano-TiO_2_ in the presence of UV.

The viability of HaCaT cells was tested following treatment with nano-TiO_2_ for 24 h (Figure 2). The low concentration of nano-TiO_2_ P25 (<50 μg/mL) was non-toxic. High concentrations (100 μg/mL) of pure anatase (A25) and rutile (R25) nano-TiO_2_ showed no significant cytotoxicity. However, exposure to 100 μg/mL P25 reduced cell viability by approximately 20% (Figure 2C). Exposure to 1000 μg/mL A25 reduced cell viability by approximately 30%, whereas exposure to 1000 μg/mL R25 reduced cell viability by approximately 20%. Exposure to 1000 μg/mL P25 reduced cell viability by approximately 45% (*p* < 0.001). The viability of HaCaT cells was decreased by nano-TiO_2_ in a concentration-dependent manner. The toxic effects of nano-TiO_2_ were related to its crystalline phase. P25, the mixture of crystalline nano-TiO_2_ phases, showed more severe cytotoxicity in comparison with the pure anatase and pure rutile samples.

To investigate the phototoxicity of nano-TiO_2_, cells were subjected to UV irradiation for one hour after treatment with nano-TiO_2_, after which cell viability was tested. There was no decrease in viability when HaCaT cells were exposed to UV irradiation alone. R25 showed no significant phototoxicity, and cell viability was similar with or without UV irradiation. However, UV light increased the damage caused by nano-TiO_2_ P25 and A25. As shown in Figure 2, P25 was more phototoxic than A25. Exposure to 50 μg/mL P25 and UV light decreased cell viability by 50% (*p* < 0.001). Exposure to 500 μg/mL A25 and UV light reduced cell viability by approximately 10% (*p* < 0.01). The mixture of crystalline phases had the highest photo-cytotoxicity. Therefore, P25, which had severe phototoxicity and lacked cytotoxic effects, was used in subsequent experiments at a concentration of 50 μg/mL.

### 3.3. The Effects of UV and Nano-TiO_2_ on α2,3- and α2,6-Linked Sialic Acids

**Figure 3:**
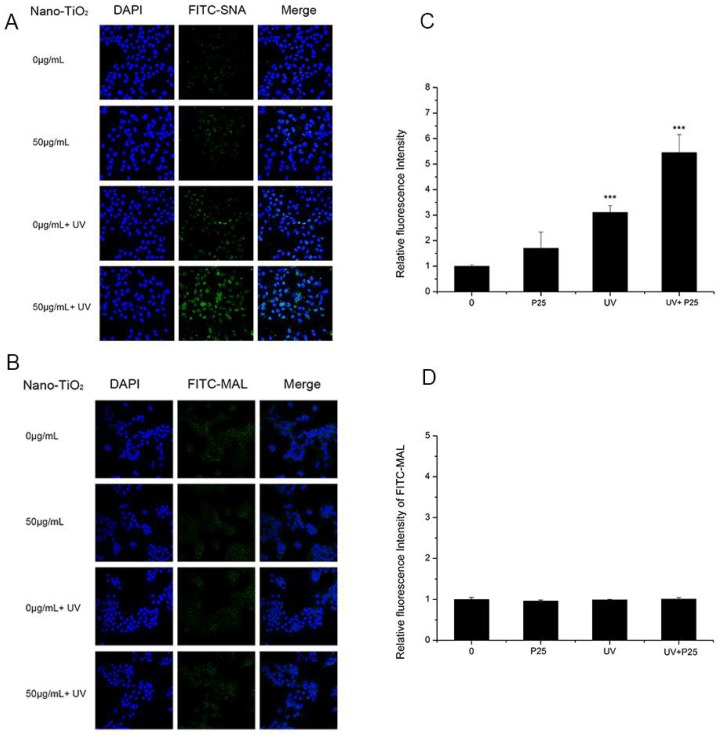
Assessment of changes in sialic acids by lectin staining. HaCaT cells were treated with 50 μg/mL nano-TiO_2_ P25, UV or the combination of nano-TiO_2_ P25 and UV. Staining was performed with fluoresceinIsothiocyanate (FITC)-labeled (**A**) SNA for α-2,6 sialic acid residues (green), (**B**) Maackia amurensis lectin (MAL) for α-2,3 sialic acid residues (green), and 4′,6-diamidino-2-phenylindole (DAPI) for nuclei (blue). The bar graphs show the fluorescent intensity analysis for (**C**) A and (**D**) B (*** *p* < 0.001, error bars are standard error of the mean).

To study whether UV and nano-TiO_2_ influence sialic acids on HaCaT cells, fluorescence-labeled lectins were detected (Figure 3). The fluorescent signal intensity of SNA (sia2-6Galβ1-4GlcNAc) on the cells treated with UV and nano-TiO_2_ P25 was significantly higher than that of the untreated cells, which indicated high expression of α2,6-linked sialic acids. In the presence of UV and nano-TiO_2_ P25 at a concentration of 50 μg/mL, the abundance of α2,6-linked sialic acids was increased by 5.9-fold in comparison with that of the control group and significantly elevated in comparison with that of the group exposed to UV treatment only and that of the group exposed to nano-TiO_2_ P25 only (*p* < 0.001). The UV group exhibited a significant increase of 2.4-fold in fluorescence intensity in comparison with that of the control group, indicating that the abundance of α2,6-linked sialic acids was increased in the UV group (*p* < 0.001). MAL-I staining for α2,3-linked sialic acids did not change significantly after any of the treatments. The fluorescent intensity analysis suggests that the groups had no significant differences in the abundance of α2,3-linked sialic acids.

**Figure 4:**
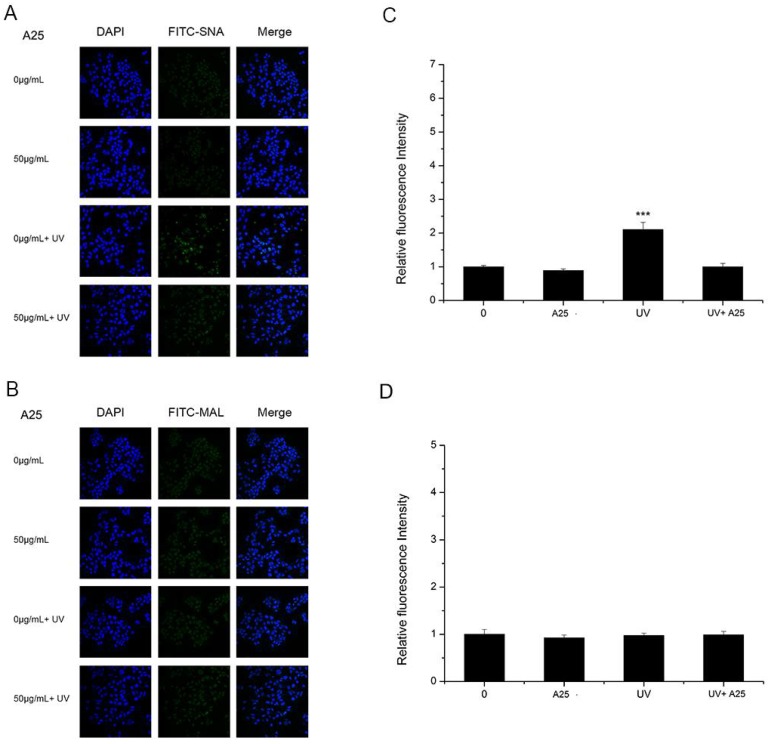
HaCaT cells were treated with 50 μg/mL nano-TiO_2_ A25, UV or the combination of nano-TiO_2_ A25 and UV. Staining was performed with FITC-labeled (**A**) SNA for α-2,6 sialic acid residues (green), (**B**) MAL for α-2,3 sialic acid residues (green), and DAPI for nuclei (blue). The bar graphs show the fluorescent intensity analysis for (**C**) A and (**D**) B (*** *p* < 0.001, error bars are standard error of the mean).

**Figure 5:**
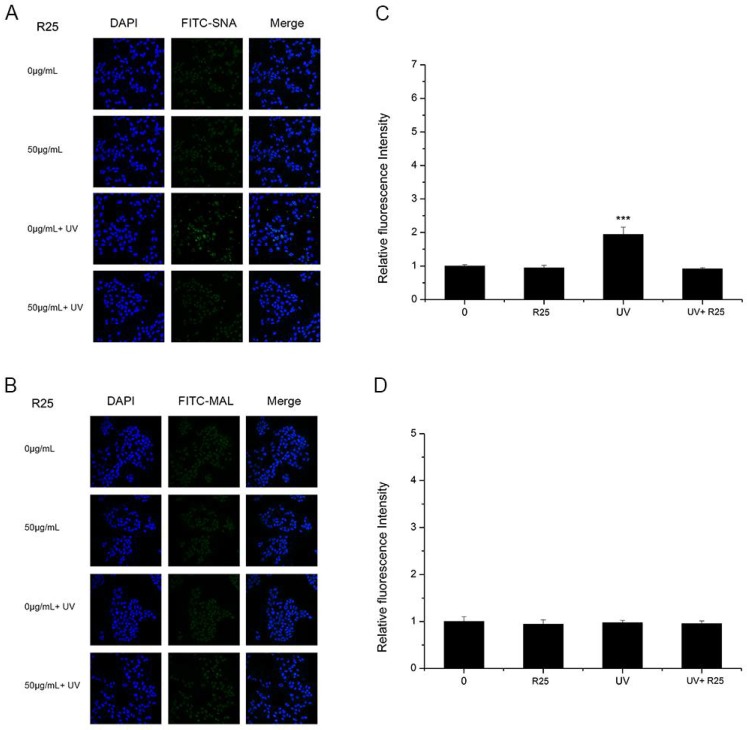
HaCaT cells were treated with 50 μg/mL nano-TiO_2_ R25, UV or the combination of nano-TiO_2_ R25 and UV. Staining was performed with FITC-labeled (**A**) Sambucus nigra agglutinin (SNA) for α-2,6 sialic acid residues (green), (**B**) MAL-I for α-2,3 sialic acid residues (green), and DAPI for nuclei (blue). The bar graphs show the fluorescent intensity analysis for (**C**) A and (**D**) B (*** *p* < 0.001, error bars are standard error of the mean).

As shown in Figure 4 and Figure 5, A25 and R25 did not alter staining for SNA or MAL-I with or without UV irradiation, which indicated that A25 and R25 did not change the abundance of sialic acids at a concentration of 50 μg/mL. These findings indicate that UV light and nano-TiO_2_ P25 can affect expression of α2,6-linked sialic acids and have no effect on expression of α2,3-linked sialic acids. 

### 3.4. Intracellular ROS Detection

**Figure 6:**
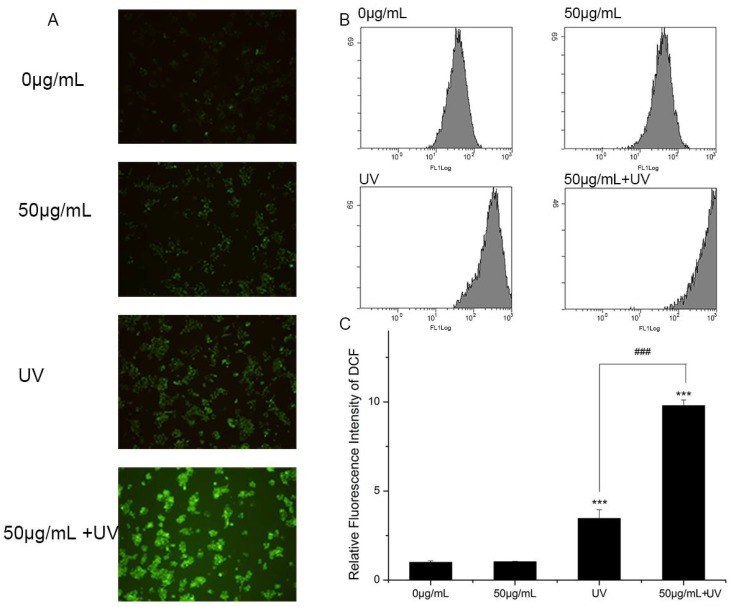
Reactive oxygen species (ROS) generation in HaCaT cells. Cells were treated with 0 or 50 μg/mL nano-TiO_2_ P25 with or without UV irradiation. After the treatment, 2′,7′-dichlorofluorescin diacetate (DCFH-DA) was imaged by fluorescence microscopy and detected by flow cytometry. (**A**) A fluorescence microscopy image, (**B**) histograms from the flow cytometry analysis, (**C**) quantification of the fluorescence intensity of 2,7-dichlorofluorescein (DCF) (mean ± SD, *n* = 3, *** *p* < 0.001, compared with the 0 μg/mL group; ### *p* < 0.001, compared with the UV group).

Although the mechanism of nano-TiO_2_ P25 toxicity was not fully deciphered, ROS was regarded as playing a critical role in the toxicity of nano-TiO_2_ P25. Therefore, ROS generation was examined using a DCFH-DA probe during treatment with UV light and nano-TiO_2_ P25. Images collected using a fluorescence microscope showed that the intracellular ROS generated in HaCaT cells was dependent on the treatment (Figure 6A). The cells exposed to nano-TiO_2_ P25 for 3 h exhibited improved intracellular ROS. The presence of UV increased ROS production compared to the absence of UV. Moreover, the cells exposed to nano-TiO_2_ P25 and UV irradiation showed the most ROS generation. Intracellular ROS were quantified by quantitative flow cytometry (Figure 6B,C). Intracellular ROS abundance was increased significantly in HaCaT cells treated with 50 μg/mL nano-TiO_2_ P25 and UV light. Intracellular ROS abundance was increased approximately 2.5-fold in cells treated with UV light alone in comparison with that of control cells and those treated with nano-TiO_2_ P25 only. A significant increase in ROS generation in HaCaT cells was detected after UV irradiation and nano-TiO_2_ P25 treatment (10-fold that of the control group, *p* < 0.001).

### 3.5. The Effect of ROS on Sialic Acid Expression

**Figure 7:**
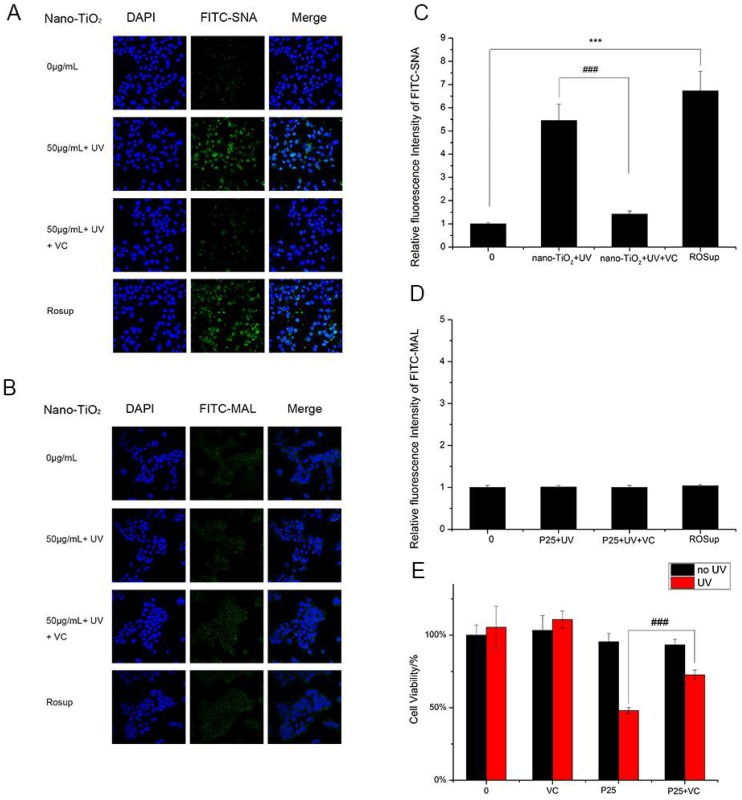
Detection of changes in sialic acids by lectin staining. HaCaT cells were treated with 50 μg/mL nano-TiO_2_ P25 and UV, the combination of 1 mM VC, nano-TiO_2_ P25 and UV, or 50 μg/mL ROSup. Staining was performed with FITC-labeled (**A**) SNA for α-2,6 sialic acid residues (green), (**B**) MAL for α-2,3 sialic acid residues (green), and DAPI for nuclei (blue). The bar graphs show the fluorescence intensity analysis for (**C**) A and (**D**) B (error bars are standard error of the mean). (**E**) The effects of vitamin C (VC) on cell viability under treatment with nano-TiO_2_ P25 and UV irradiation. Significant differences are indicated where (*n* = 5) ± SEM, *** *p* < 0.001, ### *p* < 0.001, compared with the same concentration of nano-TiO_2_ in the presence of UV without VC treatment.

The results of the analysis of ROS generation were in accordance with the results of the analysis of α2,6-linked sialic acids. HaCaT cells treated with nano-TiO_2_ P25 and UV showed enhanced ROS production and increased abundance of α2,6-linked sialic acids. These findings suggested ROS altered α2,6-linked sialic acids on the HaCaT cell surface. Vitamin C (VC), a natural antioxidant, can eliminate ROS and protect cells from oxidative damage [31]. Moreover, VC can significantly reduce intercellular ROS abundance [32]. HaCaT cells incubated with VC and nano-TiO_2_ P25 under UV irradiation showed significantly decreased binding with SNA. ROSup was used as a reagent to generate a positive control group with relatively high ROS abundance. After treatment with 50 μg/mL ROSup, a dramatic increase in SNA binding was observed. VC decreased SNA binding by approximately 5-fold in comparison with that of the group treated with UV and nano-TiO_2_ P25 (*p* < 0.001). The fluorescent intensity of the group treated with VC was similar to that of the control group. The fluorescent intensity of the ROSup group was increased by approximately 6.5-fold in comparison with that of the control group (Figure 7A,C). However, VC treatment and ROSup treatment did not alter the abundance of α2,3-linked sialic acids (Figure 7B,D). These findings indicate that P25 could lead to abnormal expression of α2,6-linked sialic acids via ROS generation. In addition, an abnormally high abundance of α2,6-linked sialic acids can be reversed by treatment with antioxidants.

HaCaT cells exposed to VC in the presence of nano-TiO_2_ P25 and UV irradiation showed increased cell viability in comparison with that of the group treated with UV and P25 (Figure 7E). In addition, VC-treated cells showed no discernible difference in viability in comparison with that of the control group. Therefore, these results demonstrate that ROS affect the level of α2,6-sialylation on the cell surface; greater ROS abundance is associated with enhanced α2,6-sialylation. Finally, exposure to VC can protect cells from nano-TiO_2_ and UV irradiation via ROS clearance.

## 4. Discussion

Nano-TiO_2_ is one of the most used nanomaterials and has thus been the subject of significant research regarding toxicity and safety. Humans can be exposed to nano-TiO_2_ during manufacturing [33]. Nano-TiO_2_ can be found in the forms of aerosols, suspensions or emulsions, which can cause toxic effects via inhalation and dermal exposure [34]. The small size and high surface-to-volume ratio of nano-TiO_2_ increase the surface energy of the particles and enhance biological reactivity [35]. ROS generated by nanoparticles can lead to cytotoxicity and genotoxicity. The skin is regularly exposed to irradiation from sunlight simultaneously with nano-TiO_2_ contained in sunscreen. Therefore, the toxicity of nano-TiO_2_ in the context of the skin is of great significance.

In this work, the cytotoxicity of nano-TiO_2_ was related to its crystalline phase, concentration, and exposure time (Figure 2). There were no significant differences in nano-TiO_2_ cytotoxicity at low concentrations of 10 μg/mL or 50 μg/mL, while nano-TiO_2_ showed cytotoxicity at concentrations of 100–1000 μg/mL. The toxic effects of nano-TiO_2_ increased in a dose-dependent manner. P25 at a low concentration of 50 μg/mL with UV light caused a 50% decrease in cell viability in comparison with that of cells exposed to nano-TiO_2_ P25 only. The cytotoxicity and phototoxicity of nano-TiO_2_ were related to its crystalline phase; P25 had greater photo-cytotoxicity in comparison with that of A25 or R25. These results are in accordance with those of previous studies, which also showed that phototoxicity was mediated by ROS generation during UVA irradiation [1,9,36]. The low concentration (less than 50 μg/mL) used for all nano-TiO_2_ treatments did not affect proliferation after 24 h of treatment, but P25 caused severe phototoxicity in the presence of UV light. Therefore, 50 μg/mL nano-TiO_2_ P25 was used for subsequent experiments.

Although the principle of nano-TiO_2_ phototoxicity is still debated, ROS are considered to be a potential mechanism for nano-TiO_2_ phototoxicity. In this study, DCFH-DA was used to estimate ROS generation. Significantly increased ROS abundance was observed in HaCaT cells after treatment with nano-TiO_2_ P25 under UV irradiation (Figure 6). Treatment with UV and nano-TiO_2_ together enhanced the level of ROS production in HaCaT cells by approximately 10-fold in comparison with that of the control group. Excessive ROS and reactive nitrogen species (RNS) have been reported as fundamental mechanisms underlying nanomaterial toxicity; they induced apoptotic signaling and various pathologies by disrupting intracellular redox homeostasis and inducing irreversible oxidative modifications of lipids, proteins or DNA [12]. However, during apoptosis, glycosylation changes occur with cell shrinkage, nuclear condensation and DNA disruption. Cells from different histological origins undergoing apoptosis and primary necrosis induced by TPEN and heat treatment had an α2,6-sialylated lactosaminic structure [20]. However, few studies have paid attention to the toxic effects of nanomaterials on glycans. Glycans perform functional and regulatory roles in various physiological processes. Glycan structure is defined by the expression of nucleotides in the corresponding gene and environmental factors [37]. Sialylation is an important glycosylation modification that plays an important role in cell signaling [38], cell adhesion [39], cell recognition [21], ageing, and senescence [40]. Considering the importance of sialylation, we investigated changes in sialylation on HaCaT cells treated with nano-TiO_2_ and UV irradiation. Cells treated with UV or nano-TiO_2_ P25 showed altered sialylation levels, while the groups treated with A25 or R25 showed no significant alterations in sialylation. However, only the abundance of α2,6-linked sialic acids was changed; no differences in α2,3-linked sialic acids were observed in the groups treated with P25 and UV (Figure 3). The group treated with UV and nano-TiO_2_ P25 showed a higher level of sialylation in comparison with those of the other groups, whereas the UV group and nano-TiO_2_ P25 group each showed a slight increase. The changes in sialic acid abundance on HaCaT cells followed a pattern similar to that of the changes in ROS generated by UV and nano-TiO_2_ P25, which indicated that the changes in sialic acids may be related to ROS generation.

To confirm the hypothesis that ROS are involved in changes in sialylation induced by UV and nano-TiO_2_ P25, we used vitamin C and ROSup to treat HaCaT cells, followed by detection of changes in α2,6-linked sialic acids and α2,3-linked sialic acids. The positive control ROSup group showed a significant 6-fold increase in sialylation in comparison with the control group. Vitamin C, as an antioxidant and free radical scavenger, is widely used to protect bio-membranes from peroxidative damage [31]. VC can decrease ROS abundance to inhibit cellular damage [32,41]. As shown in Figure 7, VC protected cells by reducing the abundance of intracellular ROS and decreasing the abundance of α2,6-linked sialic acids by approximately 4-fold in comparison with that of the group that was not treated with VC. However, VC did not produce a similar effect on α2,3-linked sialic acids. VC inhibited apoptosis caused by nano-TiO_2_ phototoxicity and increased cell viability by 25%. These results demonstrate that ROS generation affected the expression of α2,6-linked sialic acids, but had no effect on the expression of α2,3-linked sialic acids. In addition, VC reversed the abnormal expression of α2,6-linked sialic acids caused by nano-TiO_2_ and inhibited apoptosis. Changes in sialylation may be mediated by sialyltransferase in a manner regulated by ROS production. Overexpression of sialylated antigens has been reported to cause cancer, and our results suggest that clearing ROS with an antioxidant like VC can reduce sialic acid expression and thus reduced the risk of cancer. This study demonstrated that the effects of ROS generated by nanomaterials and UV irradiation on sialic acids may be a new mechanism of nanomaterial toxicity and provide insight into methods of enhancing the safety of nanomaterials by utilizing antioxidant agents to reduce ROS abundance. Moreover, change in sialic acids could be a marker of nanomaterial toxicity. Our study is the first to assess the influence of nano-TiO_2_ P25 on oligosaccharides and provides a foundation for research into the impact of nanomaterials and the development of new strategies for mitigating their toxicity.

## Figures and Tables

**Table 1:** Physicochemical properties of the nano-TiO_2_ used in this study.

Product	Crystalline Phase	Purity	Particle Size	Particle Specific Surface Area
Degussa P25	25% rutile/75% anatase	99.5%	21 nm	50 m^2^/g
Rutile 25	Rutile	99%	25 nm	51.02 m^2^/g
Anatase 25	Anatase	99%	25 nm	49.5 m^2^/g
